# Biomimetic Hydrogels – Tools for Regenerative Medicine, Oncology, and Understanding Medical Gas Plasma Therapy

**DOI:** 10.1002/smll.202403856

**Published:** 2025-02-05

**Authors:** Alice Martinet, Lea Miebach, Klaus‐Dieter Weltmann, Steffen Emmert, Sander Bekeschus

**Affiliations:** ^1^ Department of Dermatology and Venerology Rostock University Medical Center Strempelstr. 13 18057 Rostock Germany; ^2^ ZIK *plasmatis* Leibniz Institute for Plasma Science and Technology (INP) Felix‐Hausdorff‐Str. 2 17489 Greifswald Germany

**Keywords:** CAP, LTP, non‐thermal plasma, NTP, plasma medicine, reactive oxygen species

## Abstract

Biomimetic hydrogels enable biochemical, cell biology, and tissue‐like studies in the third dimension. Smart hydrogels are also frequently used in tissue engineering and as drug carriers for intra‐ or extracutaneous regenerative medicine. They have also been studied in bio‐sensor development, 3D cell culture, and organoid growth optimization. Yet, many hydrogel types, adjuvant components, and cross‐linking methods have emerged over decades, diversifying and complexifying such studies. Here, an evaluative overview is provided, mapping potential applications to the corresponding hydrogel tuning. Strikingly, hydrogels are ideal for studying locoregional therapy modalities, such as cold medical gas plasma technology. These partially ionized gases produce various reactive oxygen species (ROS) types along with other physico‐chemical components such as ions and electric fields, and the spatio‐temporal effects of these components delivered to diseased tissues remain largely elusive to date. Hence, this work outlines the promising applications of hydrogels in biomedical research in general and cold plasma science in particular and underlines the great potential of these smart scaffolds for current and future research and therapy.

## Graphical abstract

Hydrogels are receiving increased attention as innovative therapeutics for, e.g., regenerative medicine, wound dressings, and drug carriers. In addition, hydrogels are key elements in novel research models as bio‐sensors and in 3D biology, which also supports unraveling spatio‐temporal effector‐delivery systems, such as medical gas plasma technology‐derived reactive species critical in redox biology and medicine approaches.

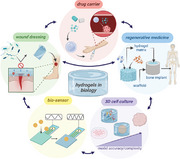

## 1 Introduction

Hydrogels are 3D polymeric networks with a vast panel of compelling properties.^[^ ^1^, ^2^ ^]^ Those hydrophilic structures can absorb a significant amount of water from synthetic or natural sources. Most are biocompatible and biodegradable; therefore, they represent a material of great interest for biological applications.^[^ ^3^, ^4^ ^]^ Notwithstanding, choosing one hydrogel can become quite a hassle among the long list. Several criteria have been identified in previous publications to distinguish and classify them, notably cross‐linking,^[^ ^5^ ^]^ physical and chemical properties, sources, and even more.^[^ ^6^, ^7^ ^]^ Besides the inherent properties of those gels, improvements have been made possible thanks to hybridization, fabrication methods, or incorporated additives within the gels, making them even more polyvalent but also remarkably specific.^[^ ^8^ ^]^ Over the past years, methods involving hydrogels have increased and continue to be developed, especially in medicine.^[^ ^9^ ^]^ Hydrogels are used nowadays as plasters or contact lenses for drug delivery tissue engineering.^[^ ^4^ ^]^ In research, two and 3D cell culture is achieved using hydrogels.^[^ ^10^, ^11^ ^]^ Moreover, hydrogel‐based biosensors have also been developed to bridge biological substances and physicochemical signals.^[^ ^12^ ^]^ Concerning this, advances in material sciences have defined smart hydrogels as dynamic scaffolds able to modify their intrinsic properties under various chemical, physical, or biochemical stimuli. This powerful feature further extended the potential scope of hydrogel applications.^[^ ^13^, ^14^, ^15^ ^]^

In another domain, physical plasma is an exciting state of matter that has been extensively studied over the past decades.^[^ ^16^ ^]^ Physical plasma, the fourth state of matter, designates an ionized gas characterized by a substantial amount of charged particles, ions, and electrons. While physical plasmas are ubiquitous in nature, they can also be artificially generated by, e.g., applying a strong electromagnetic field. Cold physical plasma, in the context of medical applications, is a type of plasma operating within a temperature range compatible with living organisms, thus, without risking severe damage.^[^ ^17^ ^]^ Generally, those plasma are ignited at atmospheric pressure using inert gases like argon, helium, or ambient air. Set in the field of applied redox biology, gas plasmas elicit their effects by generating an exceptional variety of reactive oxygen and nitrogen species (ROS, RNS) simultaneously.^[^ ^18^ ^]^ With the emergence of various plasma technologies, the development of plasma sources and devices for biology was greatly enhanced.^[^ ^19^, ^20^ ^]^ While being initially accredited for chronic wound care in dermatology, intense research efforts were made in many other medical application areas, such as oncology,^[^ ^21^, ^22^ ^]^ gynecology,^[^ ^23^, ^24^ ^]^ dentistry,^[^ ^25^, ^26^ ^]^ ophthalmology,^[^ ^27^, ^28^ ^]^ and its use as a hemostatic agent.^[^ ^29^, ^30^ ^]^ Medical gas plasma‐based instruments for chronic wound healing are becoming more and more standardized in clinics^[^ ^31^, ^32^ ^]^ while being continued to be studied in basic research.^[^ ^33^, ^34^ ^]^ The high compatibility of hydrogels and plasma physical and chemical features and the complementary needs expressed by research on both biomaterial for medical applications and plasma medicine have made plasma‐enhanced hydrogels valuable tools for the development of new reactive species carriers^[^ ^35^ ^]^ and appointed hydrogels as surrogate models to study plasma‐tissue investigations.^[^ ^36^ ^]^ This review aims to summarize recurrent hydrogels used in the field of biology with a focus on medical applications, including their advantages and drawbacks. From there on, the usage of such material to investigate the effects of cold physical plasma in biology will be discussed by depicting the numerous combination possibilities between plasma and hydrogels, illustrating either plasma‐tissue investigations using hydrogels as 3D bio‐matrix, plasma‐enhanced hydrogels, or synergetic therapies with an overview on promising approaches.

## 2 Classification of Hydrogels for Biological Applications: Advantages and Disadvantages

**Figure 1:**
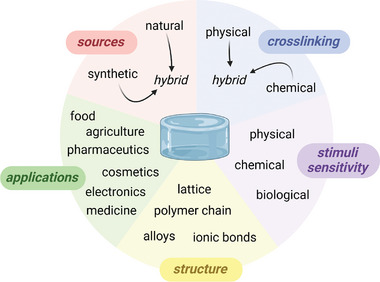
Hydrogel classifications by sources, cross‐linking methods, stimuli sensitivities, structures, and applications. Copyright: the authors.

As there are numerous types of hydrogels, there are many ways to classify them^[^ ^37^, ^38^ ^]^ (**Figure** **1**). A common distinction is made between natural and synthetic hydrogels due to significant intrinsic characteristic differences leading to divergent fields of application. Natural hydrogels are available naturally in living beings, either from animals or plants. They are known for their inherent biocompatibility and biodegradability. Moreover, they are most likely cheaper than their synthetic peers because of their natural abundance. However, the extraction process cost has to be taken into account. Synthetic hydrogels are artificially produced and well‐known for their strong mechanical properties, unlike natural ones. Their biocompatibility and degradability vary according to the structure, the polymer chains, or the cross‐linking methods.^[^ ^5^ ^]^ To optimize those gels and get the best of both gels, countless combinations of natural and/or synthetic hydrogels have been developed over the years.^[^ ^39^, ^40^, ^41^ ^]^ Methods exist to modify matrix properties and sensitivity to specific stimuli such as pH or temperature.^[^ ^42^ ^]^ Moreover, many additives can be added to the structure, such as arginyl glycyl aspartic acid (RGD) peptides, to enhance cell adhesion.^[^ ^43^ ^]^ Vice versa, using biocompatible hydrogel as a carrier for non‐biological material is also effective. The increasing complexity of those matrices with unique properties makes hydrogels ideal for specialized applications. If hydrogels are applied to living beings, they have to be biocompatible and non‐toxic for apparent reasons, and the material's biodegradability is just as important. The evolution of the structure over time in prolonged contact with living organisms, injected or grafted, is a critical question. The biodegradability of a material is defined by the aptitude to be genuinely degraded and discarded by the organism. Proteins in mammalian organisms can naturally biodegrade collagen.^[^ ^44^ ^]^ In the case of healing processes or grafts, adequate biodegradability is when the material degrades at the same pace as the natural tissue it was meant to imitate.^[^ ^8^ ^]^ When injected for drug delivery,^[^ ^45^ ^]^ once the substance is delivered, the organism should be able to evacuate, leaving as few residues as possible to avoid an immune response from the host immune system. Reversible cross‐linkable hydrogel, such as gelatin, could be advantageous in this case. The hydrogels presented below are the most common and/or bases for other hydrogels in the literature. Nevertheless, the list is not exhaustive.

### 2.1 Natural Hydrogels

Natural hydrogels (**Table** **1**) present many assets such as low toxicity, biodegradability, biocompatibility, or even low cost in most cases due to their high availability in nature. On the other hand, natural gels are most likely mechanically weak, which limits their use alone in dynamic settings.^[^ ^46^, ^47^ ^]^ Polysaccharides like dextran, starch, alginate, chitosan, and many others represent a large part of the natural hydrogels;^[^ ^8^ ^]^ however, the most common hydrogels found in studies related to medicine are protein‐base gel collagen and gelatin.^[^ ^48^ ^]^ Collagen‐based gels are most likely preferred for translational research due to the significant presence of collagen in human tissue matrices. Animal‐sourced extra‐cellular matrices (ECM) generally offer more favorable sites for cell adhesion and allow protein adsorption.^[^ ^49^, ^50^ ^]^

**Table 1:** Natural hydrogels. Copyright: the authors.

Hydrogel	Usage	Sources	Cross‐linking	Pros	Cons	Refs.
Collagen	Cellular growth matrix, regenerative medicine, drug delivery	Extracellular matrix protein, most abundant protein in mammalian organisms (29 types identified)	Thermal or chemical (pH)	Readily available (animals), biodegradable (via collagenase), biocompatible, cell signaling domains, cytocompatible, reversible gelation	High cost for a natural hydrogel, pH and temperature sensitivity, batch‐to‐batch variability, mechanically weak, limited long‐term stability, Immunogenicity	[8, 44, 125]
Gelatin	3D cell culture, drug delivery, tissue engineering, wound dressing	Extracellular matrix protein	Thermal	Non‐immunogenic, biocompatible, biodegradable, hydrophilic, reversible gelation	pH‐responsive swelling	[8, 216, 217]
Lignin	Biosensors, food industry, pharmaceutics	Plant cell walls	–	Availability, biocompatibility, eco‐friendliness, low toxicity, enzymatic degradation sensitivity, low cost	Usually needs a combination with another polymer	[218]
Fibrin	Wound healing, drug delivery, tissue engineering	Human plasma	Enzymatic	Biodegradable (fibrinolytic system), tunable viscoelasticity	Possible immune response, possible infectious transmission	[8, 219]
Hyaluronic Acid	Naturally present in wounds, joints, and brain ECM, tissue engineering, regenerative medicine, cell culture	Bacterial fermentation or animal tissues	Chemical	Binding receptor, biodegradability, biocompatibility	–	[8, 97, 220, 221, 222, 223]
Cellulose	Blood purification membrane, tissue engineering	Bacteria, plants	Thermal	Most abundant natural biopolymer, biocompatible, recyclable, thermoresponsive, durability, good mechanical strength	Not dissolving in water	[8, 97, 224, 225, 226]
Agarose	Tissue engineering, drug delivery, bio‐active coating	Marine algae	Thermal	Reversible gelation, good mechanical properties, tunable	Low degradation rate, slow adsorption/desorption	[227, 228]
Starch	Wound dressing	–	Radical polymerization	Low cost, biocompatibility, non‐toxic, hydrophilic, biodegradability, availability, pH‐sensitive, easy preparation	Use of native starch not feasible, poor mechanical properties	[225, 229]
Dextran	Site‐specific drug delivery (embedded fluorescent probe)	Bacteria	Chemical o or physical cross‐linking	Biodegradability, biocompatibility, low toxicity, low cost, highly water soluble	–	[8, 59, 230]
Alginate	Food industry, bio‐engineering, drug delivery, wound dressing, 3D cell culture, pharmaceutics	Brown seaweed, bacteria	Ionic interactions cross‐linking (Ca^2+^)	Non‐toxic, cheap, biocompatible, non‐immunogenic, availability, pH sensitivity, printable	Decrease of mechanical properties over time, need for adjuvant for cell culture (RGD), need for covalent cross‐linking to improve strength	[8, 43, 59, 97, 125, 231]
Chitosan	Drug delivery, agriculture, wound healing	Derived from Chitin	pH increasing, photo‐cross‐linking, ionic interactions, cross‐linking	Non‐toxic, heterogeneous, biodegradable (lysozyme), biocompatible, cheap, high availability	Low solubility in aqueous solution (above pH 5), weak mechanical properties	[8, 59, 97, 125, 232]
Carrageenan	Drug delivery, tissue engineering, pharmaceutics, wound healing	Seaweed	Ionic interactions	Tunable and temperature‐dependent viscosity	Physiologically unstable	[8, 233]
Chitin	Cosmetics, agriculture, drug delivery, tissue engineering scaffolds, wound dressing, cancer diagnosis	Insects, fish, seafood, plants	Enzymatic cross‐linking, fermentation	Abundant in nature, low toxicity, biodegradability, bioactivity, hydrophobicity	Low solubility in common solvents, insoluble in most organic solvents	[8, 97, 234]
Pectin	Pharmaceutics, drug delivery	Plant cell walls	Ionic cross‐linking	Biodegradable, non‐toxic, biocompatible, low cost, easy preparation, pH‐sensitive cross‐linking	Premature drug release, low mechanical properties, low drug loading	[8, 97, 235]
Pullulan	Food industry, wound dressing	Fungus	Fermentation, chemical cross‐linking	Low toxicity, highly soluble in water, biodegradable, non‐immunogenic, high water retention, antimicrobial	Poor mechanical properties, high cost	[236]

### 2.2 Synthetic Hydrogels

Synthetic hydrogels (**Table** **2**) are known for their robustness and their intensive use in pharmaceutical^[^ ^51^, ^52^ ^]^ and cosmetic industries.^[^ ^53^, ^54^, ^55^ ^]^ However, despite fine tailoring, their low biocompatibility or biodegradability makes them challenging to implement alone for biomedical applications.^[^ ^4^, ^56^ ^]^ Nevertheless, their extensive use in industry has provided substantial studies, making their use accessible from the literature. Additionally, synthesizing such scaffolds has been highly controllable and reproducible.^[^ ^56^, ^57^ ^]^

**Table 2:** Synthetic hydrogels. Copyright: the authors.

Hydrogel type	Usage	Cross‐linking	Pros	Cons	Refs.
Polyethylene Glycol (PEG)	Tissue engineering (bone prostheses, wound healing), drug delivery	Covalent cross‐linking, photo‐polymerization	Most widely used/studied for biomedical applications, enhanced biocompatibility (prevents protein adsorption), water‐soluble, biodegradable, hydrophilic, non‐toxic, non‐immunogenic	–	[8, 55, 237]
Polyvinyl alchol (PVA)	Tissue engineering (cell adhesion, artificial grafts), wound dressing	Repeated freezing and thawing, gamma irradiation, electron beam, chemical (sulfuric acid or methanol acetal), temperature	Stable, elastic, biocompatible (if physical cross‐linking), hydrophilic, biodegradable, transparent, excellent mechanical strength	Toxic for chemical cross‐linking (cross‐linker residues)	[55, 59, 238]
Polyethylene Glycol Diacrylate (PEGDA)	Wound dressing, drug delivery, Bio‐sensors, 3D bioprinting	Electron beam, photo‐cross‐linking	Hydrophilic, tunable strength, extracellular matrix mimicking, biocompatibility, non‐toxic	–	[8, 55, 239, 240]
Poly(hydroxyethyl methacrylate) (PHEMA)	Contact lenses, drug delivery, tissue engineering (e.g., artificial cornea)	Photo‐cross‐linking, chemical	Good mechanical properties, good optical transparency, water stability, non‐toxic, biocompatible, high water absorbing capacity, tunable porosity	–	[8, 55, 241]
Polyacrylic acid (PAA)	Biomedical applications	Temperature	Good adhesive characteristics, pH‐dependent swelling	–	[55, 242]
Poly acrylamide (PAAm)	Drug delivery	Chemical	Tunable mechanical properties, standardized material for cell culture, fine stiffness control, pH and temperature sensitive	2D cell culture only, no encapsulation, no cell receptor interactions, cross‐linker, toxicity	[8, 55, 242]
Polyvinyl pyrrolidone (PVP)	Drug delivery, wound dressing	Chemical, gamma irradiation, electron beam, UV‐radiation, freezing‐thawing	Transparency, biocompatibility, easily blendable with other polymers, water‐soluble, biodegradable, hydrophilic	Chemical cross‐linker, toxicity	[55, 59, 60]

### 2.3 Hybrid Hydrogels

As described above, natural and synthetic polymers are complex to implement as a simple formula. To overcome mechanical weakness and low biocompatibility, most studies have reported using and developing hybrid hydrogels.^[^ ^4^, ^39^, ^40^, ^41^ ^]^ Over the past years, more complex biologically enriched structures have emerged with very specialized applications. Options are nearly infinite, and studies on single‐component hydrogels are rare nowadays. Combinations of polymers aim for a fair compromise between strength, stiffness, biocompatibility, and hydration.^[^ ^58^ ^]^ In some cases, combinations between only natural hydrogels^[^ ^8^ ^]^ or only synthetic^[^ ^8^, ^59^, ^60^ ^]^ are also described. All those gels are used for equivalent purposes like tissue engineering, drug delivery, and others.^[^ ^45^, ^61^ ^]^ Their differences reside mainly in the cross‐linking processes and stimuli sensitivity, significantly impacting the pros and cons and usage circumstances.^[^ ^62^ ^]^ Due to their sophisticated manufacturing and low availability, hybrid hydrogels are known for being very pricey. One example is the patented matrigel, a protein‐enriched extracellular matrix favorable for tumor cell growth.^[^ ^63^ ^]^ Although criticized and despite alternative suggestions,^[^ ^64^, ^65^ ^]^ this matrix has been used for numerous studies, and many support this model for cell culture.^[^ ^66^ ^]^ Finally, the arrival of 3D printing has brought a new dimension to the use of hydrogel. Initially used for prototyping (mostly with PLA), this technology has become very popular and more affordable, allowing the use of various raw materials. Nowadays, 3D printing hydrogel scaffolds with high precision and complex patterns for advanced mechanical purposes have been reported.^[^ ^67^, ^68^, ^69^, ^70^ ^]^ This method suggests developing highly customizable scaffolds adapted to each patient.

## 3 Applications of Hydrogels and Motivations

**Figure 2:**
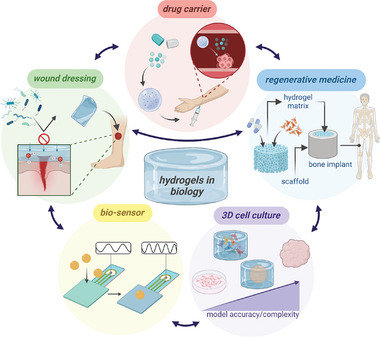
Hydrogels are used for biomedical applications such as drug carriers, regenerative medicine, 3D cell culture, bio‐sensors, wound dressing, and interconnected usages (arrows). Copyright: the authors.

Hydrogel applications in biology and medicine cross at many points (**Figure** **2**). Hydrogel drug delivery and 3D culture research benefit wound healing and regenerative medicine. Combining 3D cell culture with biosensor technologies presents exciting perspectives.^[^ ^71^, ^72^ ^]^ Lab‐on‐chip systems meet general medicine for bedside testing prospects. The synchronized use of biosensors for wound healing has been investigated using smart scaffolds.^[^ ^73^, ^74^ ^]^

### 3.1 Wound Healing

**Figure 3:**
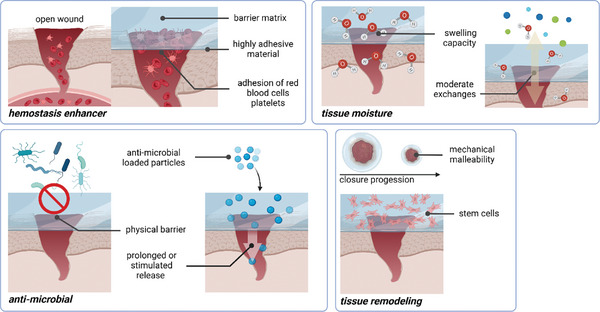
Hydrogels are used as active plasters that enhance hemostasis and have an anti‐microbial effect, control tissue moisture and exchange with the environment, and favor tissue remodeling. Copyright: the authors.

Wound healing is a complex process in which the body has to go through four phases: hemostasis, inflammation, proliferation, and remodeling.^[^ ^75^, ^76^ ^]^ During those phases and under certain circumstances, external assistance can be provided to help the body to regenerate and heal. Traditional dressings (e.g., bandages) are less flexible, have limited efficiency in extending the healing process, and are more painful, with a lower percentage of recovery.^[^ ^77^, ^78^ ^]^ Various methods have been developed, including hydrogel wound dressings.^[^ ^79^, ^80^ ^]^ In the hemostatic phase, hydrogel‐based dressings are employed first to promote hemostasis. Hydrogels can favor coagulation by forming a highly adhesive network barrier and allowing platelet adhesion onto its surface.^[^ ^81^, ^82^ ^]^ Biocompatibility of the hydrogel assures good stability, avoids undesired responses, and keeps the tissue moist while physically preventing the injury from contamination and infection^[^ ^83^ ^]^ (**Figure** **3**). Combined with an antibacterial agent (e.g., Ag^+^), hydrogel dressings can significantly reduce the healing time, inducing early inflammation and remaining harmless to the healthy tissue.^[^ ^79^, ^84^ ^]^ In the remodeling phase, stem cell‐loaded hydrogels may promote the re‐growth of the epidermal layer.^[^ ^85^ ^]^ Solid, liquid, and gaseous exchanges are made possible by the dressings' porous nature and swelling capacity,^[^ ^86^ ^]^ favoring re‐epithelization.^[^ ^87^ ^]^ The type of hydrogel employed depends on the wound's healing state and the kind of wound, varying the need for an active recovery.^[^ ^87^ ^]^ For instance, burn wounds with varying degrees and a high need for re‐epithelization can be enhanced by controlled moisture,^[^ ^86^, ^87^, ^88^ ^]^ while auto‐degrading scaffolds enable intracutaneous applications in the context of surgical wounds.^[^ ^89^ ^]^ In general, acute wound dressing should combine high adhesive and stretching abilities.^[^ ^4^, ^77^, ^90^ ^]^ Visual healing indicators like embedding a pH indicator can also be relevant.^[^ ^91^, ^92^ ^]^ Despite the enormous progress made by modern medicine over the past decades, it is well known that chronic wounds are still a worldwide disaster. They are characterized by an inability to heal, causing pain,^[^ ^76^ ^]^ requiring amputations, or indirectly contributing to lethality in 28% of cases within two years.^[^ ^93^ ^]^ Many methods have been implemented, including using several hydrogels as functional wound dressing, reported.^[^ ^77^, ^94^, ^95^ ^]^ Global outcomes revealed a high efficiency for wound healing, even in diabetic patients. Despite drawbacks such as discomfort or irritations, those results revealed promising prospects for those bio‐materials.

### 3.2 Drug/Material Delivery

**Figure 4:**
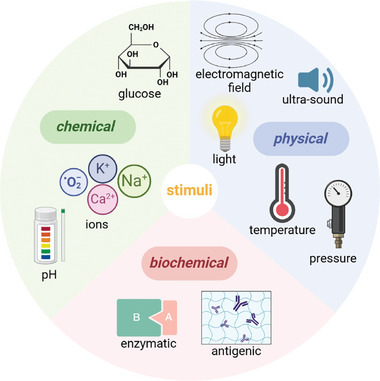
Stimuli classifications for hydrogel activation are categorized as chemical, physical, and biochemical. Copyright: the authors.

Many hydrogels are considered for internal and external drug delivery. External delivery has been described in the wound healing section above. For internal use, drug‐loaded hydrogels are injected^[^ ^45^, ^96^ ^]^ or placed and designed to deliver an adequate amount of medication to the correct location during a definite time before degradation or removal. The human body is a very complex environment, so developing a suitable material can become intricate. The need for stimuli‐responsive hydrogels is crucial to reach a specific target in the body. Possible stimuli are chemical (e.g., ionic strength, glucose, pH), biological (e.g., enzymatic, antigenic), and physical (e.g., temperature, light, ultrasound, electric and magnetic fields, pressure)^[^ ^97^, ^98^ ^]^ (**Figure** **4**). Developing a specialized hydrogel with smart drug release features is then based on those stimuli individually or combined. Combined stimuli‐sensitive hydrogels ensure high specificity of the release.

**Figure 5:**
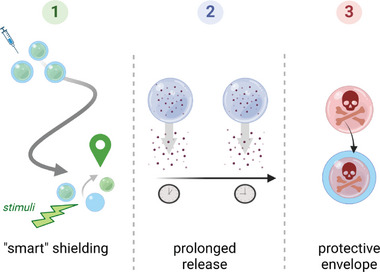
Particle embedding in hydrogels allows “smart” shielding for targeted release by external stimuli 1), offers the prolonged release of particles 2), protects the surroundings from potentially harmful compounds, or prevents the compounds from external degradation 3). Copyright: the authors.

The encapsulation of the drug or material is achieved for three main reasons. The drug could be highly sensitive or reactive and could not reach the area of interest without being degraded upstream. In case of injection at a great distance from the target to facilitate the implantation in clinics, the matrix assures the integrity of the load. Hydrogel is comparable to a sponge and is capable of releasing the substance at a low pace, ensuring a treatment over the long term and, therefore, better efficiency on resistant pathologies,^[^ ^99^ ^]^ also limiting the injection frequency. Finally, suppose the embedded material is toxic, non‐biocompatible, or insoluble in aqueous solutions. In that case, the hydrogel protects the surrounding tissues, avoids immune response or cytotoxicity, and helps its diffusion^[^ ^8^, ^100^, ^101^, ^102^ ^]^ (**Figure** **5**).

### 3.3 Regenerative Medicine

Regenerative medicine uses enriched scaffolds (e.g., cells, proteins) to mimic the original matrix and surrogate when the system needs to restore itself or as a permanent solution. This method is investigated for bones,^[^ ^103^ ^]^ skin,^[^ ^104^ ^]^ muscle,^[^ ^105^ ^]^ vascular networks,^[^ ^106^ ^]^ or internal organs.^[^ ^71^, ^92^ ^]^ Hydrogel is crucial as it contains the biological material for reconstruction and is inserted in the solid scaffold, at least for bone implants.^[^ ^103^ ^]^ Thus, it has to adhere fully to the enveloping part. In the case of skin or organ regeneration, it has to be physically robust to maintain its position in the healing area.^[^ ^107^, ^108^, ^109^ ^]^ For this purpose, one solution is to combine two different hydrogels to obtain a robust, although biocompatible, scaffold. Hybrid gel such as methacrylate gelatin (GelMA) enriched with gold nanoparticles showed advanced mechanical properties and enhanced cell viability while decreasing cost by replacing proteins (BMP‐2) with gold nanoparticles in the case of bone repair.^[^ ^110^ ^]^ Collagen/polyvinylalcohol/HA has also shown promising results for bone repair.^[^ ^111^ ^]^ GelMA+gelatin+HA bio‐printed gels for blood vessel constructs were successfully demonstrated, allowing the potential even for more complex structures.^[^ ^39^, ^92^, ^112^ ^]^

### 3.4 Hydrogel‐Based Bio‐Sensors

The rise of recent technologies, particularly electronics, has brought to the general public many tools for day‐to‐day life improvement. Within those advances, biosensors and lab‐on‐chip technologies are gradually impending. Hydrogels are a material of choice for such applications as they can perceive stimuli and trigger a reaction.^[^ ^113^ ^]^ This response is sampled, transmitted, and then analyzed. Stimuli can be biological, chemical, or physical, as reported in the drug delivery section. One major drawback would be the longevity/reusability of such apparatus for both in vivo biosensors or a lab‐on‐chip device.^[^ ^12^ ^]^ On this matter, a report was made giving the concept of a self‐cleaning membrane proposed in 2016 that would allow up to six consecutive usages of a thermo‐sensitive glucose diffuser.^[^ ^114^ ^]^ However, despite the great potential, the conclusion stated a general “proof of concept state” in the domain of biosensors. In addition, organs‐on‐chips can also be considered bio‐sensors. This technology aims to recreate the in‐vivo behavior of cells in an in‐vitro hydrogel model.^[^ ^115^, ^116^ ^]^

### 3.5 3D Cell Culture

3D cell culture consists of the entrapment of cells in a hydrogel matrix. Hydrogel is required to ensure the best survival rate of the cells. The choice of the gel has to be in line with the cell type and the experiment scheme.^[^ ^117^, ^118^, ^119^, ^120^ ^]^ Investigations of toxicity, migration, drug screening, targeted antibodies, tumor immune interaction, or tumor‐stroma interaction are potential applications.^[^ ^121^ ^]^ Analysis such as fluorescent or 3D imaging can be conducted through the transparent gel. Furthermore, one asset of collagen (or other gels, e.g., gelatin,^[^ ^122^ ^]^ is its reversible gelation over temperature or pH. Thus, cells can be released, e.g., flow cytometry. 3D cell culture offers a more realistic model for cell behavior than 2D in liquid, while unlike veritable tissues, this model remains controllable and solvable. After comparing 3D and 2D melanoma cell cultures with an in vivo reference, it was proven that cell gene expression in 3D cell culture was closer to the in vivo reference, conferring a better chemotherapy treatment resistance.^[^ ^123^ ^]^ In addition, observations under an SEM concluded a closer morphology resemblance for the 3D cultured populations. Finally, just as 2D cultures, co‐cultures or spheroids are also feasible in 3D matrices,^[^ ^124^ ^]^ along with further possibilities. This method is highly beneficial for research in cellular biology, regenerative medicine, wound healing, and biosensors. In the long term, studies predicted a potential replacement of 2D culture by 3D culture.^[^ ^125^ ^]^

## 4 The Use of Hydrogels in Biology and Plasma Research

**Figure 6:**
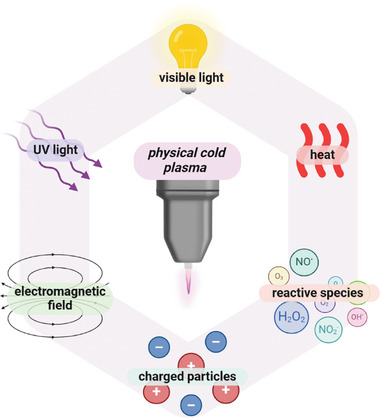
Properties of cold physical plasma: plasma is composed of reactive species and charged particles, exudes heat, and radiates electromagnetic fields, UV, and visible light. Copyright: the authors.

**Figure 7:**
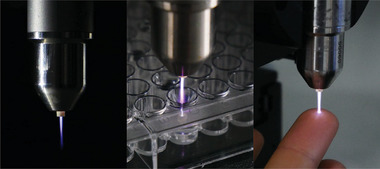
Representative images of operating the kINPen plasma device in air and conductive set‐up. Copyright: the authors.

In many ways, the crossing of hydrogel with plasma becomes increasingly investigated (**Table** **3**). The combined use of hydrogels and plasma is supported by the sensitivity of hydrogels under numerous stimuli highly compatible with plasma attributes (Figure 4). Through its inherent properties such as electromagnetic fields, reactive species, UV radiation, or heat^[^ ^126^ ^]^ (**Figure** **6**), physical plasma can enhance the polymerization process of hydrogels and introduce new biological, chemical, and physical properties to the gel structures.^[^ ^127^ ^]^ Reactive oxygen and nitrogen species (here abbreviated as ROS because most RNS also contain reactive oxygen) can modify the intrinsic gel features and act as dopants that can be released in prolonged timescales. Plasma devices for biological applications usually operate in the ambient air or with an inert gas such as argon or helium at atmospheric pressure and low temperature. Studies also investigate gas admixtures to the carrying gas like oxygen or nitrogen to enhance particular reactive species production.^[^ ^128^, ^129^, ^130^, ^131^ ^]^ Device designs vary according to the use of plasma jets like the kINPen^[^ ^132^ ^]^ (**Figure** **7**) to classic DBD with planar electrodes;^[^ ^133^ ^]^ therefore, different plasma chemistries are involved. Specific devices such as DBD patches for single plasma treatment^[^ ^134^ ^]^ or Printed Circuit Board (PCB) electrodes^[^ ^135^ ^]^ are also reported. In the case of pure material synthesis, the plasma process at low pressure is described to, e.g., ignite an N_2_‐NH_3_ plasma to treat hybrid hydrogel (CMGG/PVA).^[^ ^136^ ^]^

**Table 3:** Applications of hydrogels combined with plasma. Copyright: the authors.

Category	Main field	Hydrogel type	Hydrogel adjuvant	Plasma source (*carrier gas*)	Main findings	Refs.
ROS carrier	Antimicrobial	Acryloyldimethylammonium taurate/VP copolymer (AVC), hydroxyethyl cellulose (HEC), Carbomer 940 (Carbomer)	Plasma‐treated water, ROS, scavengers	S‐DBD (Surface dielectric barrier discharge) (ambient air)	Comparison of indirect (plasma‐treated water for dissolution), direct plasma‐treated hydrogels, and plasma‐treated water	[41]
ROS carrier	Cancer	Pluronic hydrogel	Immune checkpoint blockade (ICB)	Plasma jet	Combination of cold physical plasma and ICB inhibited tumor growth and metastatic spread	[144]
ROS carrier	Cancer, skin disease	Aristoflex AVC hydrogel (Ammonium Acryloyl Dimethyltaurate / VP copolymer) (AVC)	none	DBD (ambient air)	Comparison between plasma‐treated water and hydrogels; prolonged ROS release found with hydrogel	[99]
ROS carrier	Cancer	Alginate	Fluorescent probes	kINPen (argon), helium needle	Alginate was found to be a suitable vehicle for ROS; the cross‐linking process degraded ROS storage capacity	[137]
ROS carrier	Cancer	Gelatin	Griess reagent	kINPen (argon), helium needle	Assessment of ROS carrier capacity and delivery compared to plasma‐treated water	[143]
ROS carrier	Antifungal (wound dressing)	Polyacrylamide (PAAm) hydrogel	Plasma‐treated water	HEDBS plasma jet (ambient air)	Comparison of plasma‐treated hydrogel and water: lower oxidation‐reduction potential and longer antifungal lifetime of hydrogels than plasma‐treated water	[140]
ROS carrier	Cancer	Methylcellulose	AUR, Coumarin, Griess reagent	kINPen (argon)	Development of temperature‐sensitive gel; gelation at body temperature; liquid at room temperature; prolonged ROS release; enhanced mechanical properties	[141]
ROS carrier	Cancer (bone)	Gelatin/alginate	–	kINPen (argon)	Ceramic bone implant infiltrated by plasma‐treated hydrogel; investigation of ROS release over treatment time	[243]
ROS carrier	Vitiligo (skin conditions)	Hydroxyethyl cellulose	Plasma‐treated water	Plasma needle (ambient air)	Comparison between plasma‐treated gel and direct treatment showed comparable improvement; successful clinical trial	[151]
ROS carrier, surface modification	Biomedical applications (cancer, anti‐infectives)	Carboxymethyl guar gum, polyvinyl alcohol (PVA)	CHR (anticancer effects)	Direct glow discharge (N_2_+NH_3_)	Treatment of hydrogel to enhance biocompatibility, biodegradability, antibacterial effect, and wettability (etching, anticancer activity, embedded CHR)	[136]
ROS carrier, core modification	Wound healing	Silk‐fibrin composite gel	Griess reagent, DCF	Plasma (N_2_, He+O_2_, N_2_+Ar)	Plasma‐induced microstructure and chemical modifications; improved mechanical properties, cell viability, and wound regeneration in mice, promoting inflammatory response	[130]
ROS carrier, core modification	Antibacterial/antifungal	Chitosan	Plasma‐treated water (tap and purified water)	Plasma jet (ambient air)	Material investigation of plasma‐treated water‐based hydrogels; microscopic morphologic alterations and higher electric conductivity or total dissolved solids and lower pH were observed with increased plasma treatment time improving antibacterial/antifungal activity and hemocompatibility	[244]
ROS carrier, surface modification	Cartilage regeneration	Printable hydrogel inks, composed of 40% w/w PEG (Mn 300), 60% w/w PEGDA (Mn 700)	Drug‐loaded nanoparticles, PLGA nanoparticles	Helium needle	Plasma‐treated 3D printed scaffolds improved hydrophilicity (surface nano‐roughness) and stem cell adhesion	[139]
ROS carrier, tissue modeling	Cancer	Collagen	Resazurin, phenol red, APF, HPF, DAF	kINPen (argon)	Investigation of ROS transport from gel to liquid; plasma‐induced pH modifications; ROS‐in‐gel diffusion assays; 3D cell culture screening in collagen	[121]
ROS carrier, core modification, tissue modeling	Bone implants	Matrigel, gelatin (substitute of matrigel), collagen	Recombinant human bone morphogenetic protein‐2, bone cells, AUR, DAF‐2, H_2_DCFDA, DMSO and horseradish peroxidase as scavengers	DBD (ambient air)	In vivo, plasma‐treated matrigel increased bone formation for µs treatment compared to control (decrease for ns treatment), increased expression of adhesion proteins, and led to activation of survival pathways	[145]
Surface modification	Tissue engineering (bone)	Poly(ethylene oxide) (PEO)	–	Plasma Stylus Noble (Ar, Ar+H_2_O)	Plasma‐assisted 3D scaffold manufacturing enhanced bioactivity by improving protein adsorption and cell adhesion onto the matrix	[245]
Surface modification	Cancer	Polyvinyl alcohol (PVA), alginate	5‐fluorouracil (5FU)	Cold HMDSO plasma deposition	Plasma‐treated surface modification enhanced hydrophobicity and prolonged drug release (smart swelling); improved mechanical properties; lower water uptake capacity; effects pH‐dependent	[246]
Surface and core modification	Biomedical applications	Poly(ethylene oxide)‐based triblock copolymer (tPEO) photo‐cross‐linkable hydrogel	–	Plasma jet (helium)	Plasma‐induced pre‐cross‐linking and enhanced mechanical properties	[247]
Core modification	Cancer, biosensors	Chitosan‐based hydrogel, nitrogen‐doped graphene quantum dots (NGQDs)	–	Micro‐plasma (ambient air)	Hydrogel synthesis by exposing chitosan to micro‐plasma	[248]
Core modification	Cancer	Chitosan‐based hydrogel, nitrogen‐doped graphene quantum dots (NGQDs)	–	Micro‐plasma (ambient air)	Plasma‐induced cross‐linking to avoid temperature and toxic cross‐linking compounds for pH‐sensitive carrier	[81]
Surface modification	Bone scaffolds	2‐hydroxyethyl methacrylate (HEMA), Poly(ethylene glycol) methacrylate (PEGMA), Hydroxyapatite (HAp) onto PLA (poly(lactic acid)) structure	–	Plasma (argon)	Plasma‐induced hydrophilicity enhanced adhesion on PLA and cells	[163]
Surface modification	Stomach infection, bacteria, food industry	Chitosan/gelatin	Amoxicillin drug	DBD (He+O_2_)	Plasma treatment enhanced hydrophilicity, mechanical properties, topographical modifications by ROS etching, and thermal denaturation, favoring prolonged drug release, antibacterial effects, pH sensitivity, and biodegradability	[156]
Surface and core modification	Bone implants	Chitosan, bioresource‐derived nitrogen‐doped GQD (NGQD)	–	Micro‐plasma (ambient air)	Plasma‐enhanced cell adhesion of implants	[103]
Surface modification	Drug carrier	Chitosan	–	–	Plasma etching induced morphology and chemical modifications, hydrophobicity, and improved antibacterial effect	[159]
Tissue modeling	Antifungal (nail)	Agarose	Bacteria, Candida albicans, and Trichophyton mentagrophytes	Single‐use DBD patch electrode (ambient air)	Direct plasma exposure reduced bacteria viability	[134]
Tissue modeling	Biomedical applications (pH)	Agarose	Fluorescein	Plasma jet (helium)	Plasma acidification of the models varied according to initial pH, treatment time, type of plasma jet capillaries, and buffer capacity, impacting the size and the intensity of pH change	[36]
Tissue modeling	Cancer	Agarose	Terephtalic acid	Plasma jet (argon)	Agarose gel as a ROS barrier suggested a modest impact of long‐lived species or electric field on cells compared to short‐lived species; OH penetration suggested (0.6 mm in the liquid)	[199]
Tissue modeling	Wound healing	Agarose	AUR, Fluorescein	DBD (ambient air)	The agarose model was compared to the rat model and found suitable for analyzing ROS penetration	[193]
Tissue modeling	Skin desinfection	Agarose	TPA, Indigo trisulfonate, TSA, sulfanilamide, N‐(1‐Naphthyl) ethylenediamine, dihydrochloride, folic acid	Surface micro‐discharge (SMD) (helium and air)	Different inactivation rates for the two gases; ROS investigation in the gel	[195]
Tissue modeling	Wound, DNA repair research	IntraSite Conformable (Smith & Nephew)	–	Plasma jet (helium)	Hydrogel to simulate epidermic tissue and assess ROS type penetration; limited presence of OH in the liquid; less reduction of H_2_O_2_	[203]
Tissue modeling	Biomedical applications, food industry	Gelatin	–	Plasma jet (helium)	Influence of protein and molecular ground state oxygen on plasma generation and transport of ROS in tissue; gelatin layer on top of liquid containing ROS‐sensitive probe (for H_2_O_2_, NO_2_^−^, OH•); conductive treatment; proteins in liquid either inhibited or enhanced ROS production (depend on the O_2_ concentration)	[249]
Tissue modeling	Biomedical applications	Agarose	KI‐starch	Plasma jet (argon)	UV photolysis promotes the generation of ROS in the tissue model surface; ROS were transported to millimeter depths via a slower molecular process	[211]
Tissue modeling	Biomedical applications	Gelatin	NaNO_2_ (simulate plasma‐assisted drug penetration)	Plasma jet (helium)	Gas flow and electric fields played a crucial role in permeability; effects of gas flow confined in the surface layer; electric field holistic; effect decreases with the increasing mass fraction of gelatin	[213]
Tissue modeling	Biomedical applications	Gelatin	KI‐starch	Plasma jet (Ar+O_2_)	O_3_ penetration depth was plasma treatment time and water content dependent; plasma‐derived ROS covered the surface with good uniformity despite the small size of the plasma jet; angled treatments	[198]
Tissue modeling	Biomedical applications	Gelatin	Griess reagent	DBD (ambient air)	ROS penetration through gelatin barrier; penetration speed of nitrite species increased with gelatin water mass fraction; low electric field reduced the barrier effect of the gelatin	[215]
Tissue modeling	Cancer	Agarose	KI‐starch	Plasma jet (helium) + shielding	Agarose gel was used to display the impact of the shielding on spatial ROS distribution	[192]
Tissue modeling	Cancer	Collagen	Lung cancer cells; Amplex red and Griess reagent	DBD (ambient air)	Lung cancer cell apoptosis was related to plasma treatment time; labeled cells to assess the depth effect in the 3D matrix (more dead cells at the surface)	[181]
Tissue modeling	Biomedical research	Agarose	KI‐starch	Plasma jet (He, He+O_2_, O_3_)	Investigation of plasma‐generated RNOS transport from liquid to gel, dependency of the height and water layer thickness	[128]
Tissue modeling	Biomedical research	Agarose	KI‐starch	Plasma jet (He, He+O_2_)	He/O_2_ produced a greater ROS area in KI‐starch compared to He plasma	[129]
Tissue modeling	Biomedical research	Agarose	KI‐starch	Plasma jet (He+O_2_)	Investigation of 2D ROS distribution through the liquid on ROS‐sensitive gel detector; use of scavenger in the liquid for specific species identification; H_2_O_2_ and O_2_^−^ were key species	[188]
Tissue modeling	Biomedical research	Agarose	KI‐starch	Plasma jet (He+O_2_)	Investigation of plasma gas flow induced vortex in ROS distribution; ROS diffusion depended on the relationship between two flows: high gas flow rate formed XY diffusion (donut shape) and low gas flow rate formed radial‐shaped oxidation pattern	[187]
Tissue modeling	Biomedical research	Agarose	KI‐starch	Plasma jet (He, He+O_2_, O_3_)	Plasma‐generated ROS transport from liquid to gel; plasma device distance was critical for the extent of ROS penetration	[250]
Tissue modeling	Biomedical research	Agarose	KI‐starch	Plasma jet (He+O_2_)	Partial supply of reactive species on the tissue model surface; ROS concentration dependency of distance and tissue model thickness	[189]
Tissue modeling	Biomedical research	Agarose	KI‐starch	Plasma jet (He, He+O_2_)	ROS penetration investigation through agarose gel	[204]
Tissue modeling	Drug delivery	Agarose	–	Flexible plasma patch (ambient air)	Transdermal drug delivery was enhanced by flexible plasma	[251]
Tissue modeling	Biomedical applications	Gelatin	KI‐starch	Plasma jet (He+O_2_)	Investigation of ROS ring‐shaped surface patterns: diameters increased with gas flow rate but were unchanged with plasma‐increased treatment times; patterns changed with inclination angle; penetration depth of O_3_ increased linearly with plasma treatment time or the water content of model tissue	[191]
Tissue modeling	Biomedical applications	Gelatin	Phospholipid vesicles (vesicles containing a self‐quenched dye)	Plasma jet (helium)	Treatment angle significantly influenced the nature and level of damage to phospholipid vesicles	[252]
Tissue modeling	Biomedical research	Agarose	KI‐starch	Plasma jet (helium)	Investigation of ROS pattern on gels; gas flow modified the surface but not the ROS diffusion profile	[253]
Tissue modeling	Biomedical applications	Agarose	–	Plasma jet (helium)	Agarose was used as a ROS barrier above a liquid (4mm); UV absorption of water in real‐time; accumulation of the ROS in agarose during exposure; time‐lapsed release of ROS; four molecules transported through agarose: H_2_O_2_, NaO_2_, HNO_3_, O_2_	[254]
Tissue modeling	Biomedical applications	Agarose	–	Plasma jet (helium)	Profile and evolution of the species present in the liquid with treated agarose ROS barrier above (1.5 mm): H_2_O_2_, NaO_2_, HNO_3_, O_2_	[255]
Tissue modeling	Biomedical research	Agarose	–	Plasma jet (helium and argon)	Investigation of species diffusion into water through agarose target compared to conducting treatment with water; significant differences in concentrations of ROS; comparison of He and Ar plasma modes	[256]
Tissue modeling	Biomedical research	Agarose	–	Plasma jet (helium)	Transport of neutral and ionic species through agarose barrier (3.2 mm) found using mass spectrometry; neutral species are unable to go through agarose; positive and negative species can go through the agarose target but with time‐lag (minutes)	[257]
Tissue modeling	Biomedical research	Agarose	KI‐starch, fluorescein	Plasma jet (helium)	Spatially resolved oxidation and pH change patterns for several plasma frequencies	[202]
Tissue modeling	Cancer	Agarose	AUR	Plasma bullet (helium)	Propagation of plasma bullet into conductive agarose tube targets; understanding possibilities for internal use with fast imaging; OES; fluorescent dye in the gel (H_2_O_2_); T and L‐shaped tubes	[194]
Tissue modeling	Biomedical research	Gelatin	KI‐starch	Plasma jet needle (He, He+O_2_, He+H_2_O)	Production and penetration of H_2_O_2_ through gelatin; scavenging of OH radicals at plasma gel interface; delay between plasma treatment and diffusion	[131]
Tissue modeling	Food industry	Gelan gum plate (Gelrite) Poloxamer gel (Pluronic F‐127)	Polyphenol oxidase (PPO) and peroxidase (POD) (enzyme responsible for browning reactions and loss sensorial or nutritional quality)	kINPen (Ar, Ar+O_2_)	Plasma reduced enzyme activity in the gel; homogeneous treatment observed; good extractability found	[258]
Tissue modeling	Cancer, wound healing	Gelatin	DCFH, OPD/HRP	Plasma jet (helium)	Plasma‐derived ROS delivery from 150 µm to 1.5 mm below the surface of the gelatin film; the control group contained H_2_O_2_ solution	[196]
Tissue modeling	Biomedical research	Gelatin, IntraSite Conformable (Smith & Nephew)	DNA, OPD‐HRP, cell model (encapsulated DNA in phospholipid vesicle synthetic cell models)	Plasma jet (helium)	H_2_O_2_ transported through the dressing; cost‐effectiveness; rapid analysis	[259]
Tissue modeling	Cancer	Agarose	–	Plasma jet (helium)	Agarose tissue surrogate as ROS barrier (varying thickness), comparison with a mouse model	[260]
Tissue modeling	Cancer	Agarose	–	Plasma jet (helium)	Agarose tissue surrogate as barrier (varying thickness and disposition)	[261]
Tissue modeling	Antifungal	Agarose	KI‐starch	PCB electrode plasma source (ambient air)	Study of a multi‐jet plasma source (PCB used as an electrode); observed the potential for large‐area treatment	[135]

### 4.1 ROS Carrier

**Figure 8:**
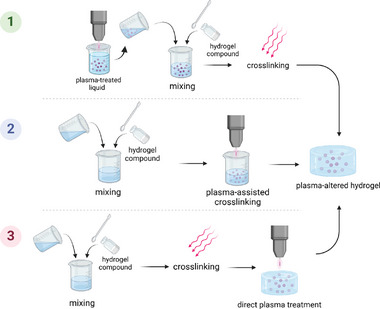
Preparation of plasma‐altered hydrogels by dissolution of hydrogel compound by plasma‐treated solution 1), plasma‐assisted cross‐linking 2), and plasma treatment of polymerized hydrogel 3). Copyright: the authors.

**Figure 9:**
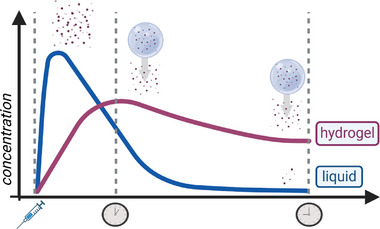
Drug‐release comparison between liquids and hydrogels. Hydrogels offer a prolonged and more steady release (purple), while liquids display a single spike release (blue). Copyright: the authors.

Hydrogels' high porosity and swelling capacity make them perfectly suitable as ROS carriers.^[^ ^41^, ^99^, ^137^ ^]^ Multiple studies have reported the possibility of using hydrogel for external or internal use.^[^ ^138^ ^]^ Three ways are identified for ROS carrier hydrogel preparation (**Figure** **8**). One consists of preparing the hydrogel with the standard, generally distilled water or phosphate‐buffered saline solution. Once solidified, the gel is then exposed to plasma.^[^ ^139^ ^]^ Another way is to use plasma during the gelation as a cross‐linker^[^ ^127^ ^]^ or with another cross‐linking method, depending on the hydrogel. The third method uses a plasma‐treated solution (e.g., water) mixed with the hydrogel base (e.g., powder). Many studies have compared the ROS release efficiency of plasma‐treated hydrogel in opposition to plasma‐treated water^[^ ^41^, ^99^, ^137^, ^138^, ^140^, ^141^ ^]^ (**Figure** **9**). A majority agreed on a higher concentration of long‐lived species after a prompt exposition of the plasma‐treated water. However, plasma‐treated hydrogel showed a more extended release capacity, exceeding the ROS concentration of the plasma‐treated water after a definite duration. The main impact of plasma‐treated hydrogel is ruled by long‐lived ROS release emerging from secondary plasma gas phase species reactions.^[^ ^142^ ^]^ Like other redox‐based therapy approaches applied in the clinic, e.g., photodynamic therapy, ROS exposure follows the hormesis concept.^[^ ^18^ ^]^ In an in vitro study, where treated gel and water were compared, the prolonged release of ROS via plasma‐treated hydrogel was shown to more efficiently induce apoptosis on cancer cells A375 without harming healthy HaCaT cells.^[^ ^99^ ^]^ This selectivity was reported in other studies.^[^ ^130^, ^143^ ^]^ Plasma‐treated hydrogel with additionally embedded immune checkpoint blockade (ICBs) was investigated as an injectable substance^[^ ^144^ ^]^ and demonstrated promising results on tumor growth and metastatic spread inhibition. Depending on the application, various plasma sources have been studied and compared, from plasma jets^[^ ^137^, ^143^ ^]^ to larger DBD devices, to identify the most effective ROS infiltration into the gel.^[^ ^145^ ^]^ Plasma‐derived ROS are not only efficient against cancer cells but were initially revealed as decisive as antifungal/antibacterial methods. At first investigated for disinfection via direct treatment for tools,^[^ ^146^ ^]^ wounds,^[^ ^147^ ^]^ or implants,^[^ ^148^ ^]^ hydrogels represent new support to carry ROS into tissues to trigger antifungal/bacterial response.^[^ ^41^ ^]^ Plasma‐treated hydrogels help obtain a more extended diffusion, improve the effects, and thus benefit more resistant pathologies than plasma‐treated water. Like standard plasma‐treated dressings,^[^ ^149^ ^]^ those hydrogels can be installed directly as wound dressing.^[^ ^150^ ^]^ They maintain the hydration of the tissue, protect it from the external environment, and enhance the healing process (Figure 4). Moreover, additional drugs can be loaded into the hydrogels to create an active scaffold.^[^ ^136^ ^]^ This method was also investigated for skin diseases such as vitiligo and showed promising results in patients,^[^ ^151^ ^]^ encouraging further dermatology applications. Besides extracutaneous use, recent studies have considered injectable hydrogels as an on‐site anticancer therapeutic. In this case, the choice of the appropriate hydrogel is central. A recent study presented a methylcellulose‐based hydrogel liquid at room temperature, ideal for plasma treatment, solidifying at body temperature,^[^ ^141^ ^]^ as well as plasma‐treated alginate‐based hydrogel, which is solidifying in water.^[^ ^142^ ^]^ Alginate can also solidify when cross‐linked with Ca2+ present in the body but can also be added to assure solid gelation,^[^ ^152^ ^]^ making it an excellent option for this purpose. Those findings highlight the interest in using hydrogel for such usage. On an anti‐cancer efficacy scale, plasma‐treated hydrogel ranked between plasma‐treated water and direct plasma treatment.^[^ ^138^ ^]^ Plasma‐treated water contains a low variety of ROS and a low treatment precision; however, it is easily applicable to all types of cancer by injection.^[^ ^153^, ^154^ ^]^ On the other hand, direct plasma treatment displays a great abundance of ROS with a high load capacity, although direct plasma use remains challenging for some internal applications. Plasma treated hydrogel is injectable yet still viscous and selective, it has a lower ROS load than direct treatment, however higher than plasma treated water. In addition, treatment of materials by plasma may induce further modifications which are discussed below.

### 4.2 Surface and Core Modifications

Tissue engineering always seeks new methods to provide the most suitable scaffolds, combining robustness and biocompatibility for regenerative medicine. With this matter, plasma surface modification purposes seem a relevant candidate. Plasma is well known for its capacity to alter surfaces, and its use has already broadened in countless applications in industrial spheres such as electronics, textiles, or manufacturing.^[^ ^155^ ^]^ The extensive use of this technology both shows its high efficacy and grants myriad studies becoming more and more sharpened to specific materials. Thanks to particles displayed in the state of plasma, it can induce the etching,^[^ ^156^, ^157^ ^]^ deposition,^[^ ^158^ ^]^ or doping of the target,^[^ ^6^ ^]^ changing surface properties and therefore promoting hydrophilicity/hydrophobicity,^[^ ^139^, ^156^, ^159^ ^]^ adhesion capacity,^[^ ^139^ ^]^ chemical reactivity, pH sensitivity,^[^ ^156^ ^]^ biocompatibility,^[^ ^139^, ^160^ ^]^ or biodegradability.^[^ ^156^ ^]^ ROS modify the bio‐surface chemistry on a long timescale observation (seconds to minutes). They bond with surface bio‐molecules, lowering the pH^[^ ^161^ ^]^ and creating an oxidative gradient between the surface and the bulk of the material. PVA gel matrix has shown drug release capacity under chemical plasma stimuli.^[^ ^162^ ^]^ In a second time, this gradient gets progressively diffused into the material to return to a balanced state. Cold physical plasma is capable of modifying the surface and applied during the gel's cross‐linking process.^[^ ^127^ ^]^ As seen in the previous part, plasma can mechanically^[^ ^156^, ^159^ ^]^ alter the hydrogel, making it more resistant and generating new chemistry.^[^ ^130^ ^]^ In the case of bone implantation, plasma treatment enhanced the bio‐properties of the gels, conferring better hydrophilicity and nano‐roughness to facilitate cell adhesion.^[^ ^103^, ^139^, ^163^, ^164^ ^]^

### 4.3 Tissue Modeling for Plasma Research

The investigation of plasma for medical application implies the need to understand the effects of physical plasma on tissues to be quantified, understood, and optimized for its application in clinics. Hydrogels are already used in many bio‐medical contexts (Figure 2) as biological matrices and offer stable support for plasma investigations and plasma‐enhanced therapies. Indeed, many studies describe hydrogels as capable of mimicking tissue‐similar properties (e.g., dielectric,^[^ ^165^, ^166^, ^167^, ^168^, ^169^, ^170^, ^171^, ^172^, ^173^, ^174^ ^]^ mechanical,^[^ ^175^ ^]^ biological,^[^ ^176^ ^]^ and acoustic^[^ ^177^ ^]^), therefore being relevant for investigating plasma effects on the biological target. However, natural tissue (ex vivo and in vivo) is the most accurate model but presents several disadvantages. It is usually quite troublesome to obtain, especially in consistent quantity, whether for harvested tissues or directly on patients or animals. In the case of ex vivo samples, it becomes unstable quickly over time. Last but not least, it constitutes a complex and sometimes overwhelming matrix in which investigating specific plasma effects is arduous. For instance, the signals from fluorescent probes for ROS assessment, especially short‐lived species, are disturbed by the tissue fluorescence background and thus make measurements and analysis much more intricate. On the other hand, 2D cell culture models have been widely investigated for plasma biological effects.^[^ ^178^ ^]^ However, cells show many differences when cultured in 2D compared to growing in 3D, as shown in.^[^ ^123^, ^179^ ^]^ Hydrogels meet both models' advantages by offering a more realistic 3D matrix with even embedded cells while being more straightforward and accessible. Moreover, the transparency of hydrogels represents a veritable asset for either 3D cell culture, allowing optical analysis such as High Content Imaging (HCI) of stained cells as well as embedding of colorimetric or fluorescent probes for ROS detection or fluorescent stained cells in a 3D matrix.^[^ ^180^, ^181^ ^]^ Moreover, the importance of conducting plasma treatment conditions to the matrix to induce cell death in 3D cancer cell culture in matrigel was demonstrated.^[^ ^182^ ^]^ In another study, the metabolic activity of the cells was successfully measured with hydrogel‐adding resazurin. Adding cells to the hydrogel helps to understand how cells in an authentic matrix would be affected by plasma treatment.

**Figure 10:**
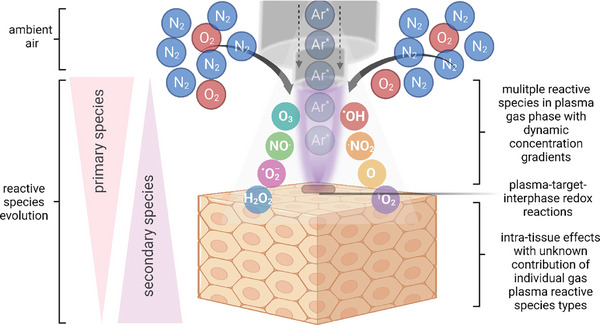
Principle of reactive species generation in the case of an atmospheric pressure argon plasma jet. Reproduced with permission.^[^ ^21^ ^]^ Copyright 2021, Elsevier.

In a related manner, one of the biggest questions in plasma medicine is how profound the impact of plasma is on tissues and its biological translation. If primary‐generated ROS^[^ ^21^, ^183^ ^]^ (**Figure** **10**) were proven not to reach the underlayer (e.g., deeper epidermis or dermis) of the skin barrier, this would explain the good tolerability of medical gas plasma technology in clinical applications. In this regard, many studies quantified the penetration depth of certain species,^[^ ^184^, ^185^ ^]^ their diffusion,^[^ ^186^ ^]^ and what they may trigger into tissues using biomimetic hydrogel models. For this purpose, ROS probes are embedded into gel matrices, and signals from the probes are quantified after plasma treatment. Numerous publications have presented the use of potassium iodide and starch in the hydrogel. The iodine ion reacts with ROS, and the chain reaction with starch turns the gel from transparent to purple, allowing the identification of the oxidized area. Potassium iodide is sensitive to several ROS, such as H_2_O_2_, OH, O_3_, HO_2_, singlet oxygen, O_2_^−^, or O.^[^ ^187^ ^]^ The selectivity of iodine does not allow the differentiation between those species. However, the reaction is immediate, and the method is easy to establish. This combination has been extensively investigated in various configurations to investigate ROS diffusion patterns in gels.^[^ ^187^, ^188^, ^189^, ^190^, ^191^, ^192^ ^]^ The ROS oxidation pattern was evaluated when running through a liquid layer before oxidizing the gel to mimic tissue moisture layers.^[^ ^129^, ^181^, ^187^, ^188^ ^]^ In those cases, height, liquid thickness, gas flow, or gas composition were significant parameters influencing the ROS pattern on the gel. In this configuration, adding scavengers into the upper liquid layer highlighted the key species going through the liquid and oxidizing the gel, respectively, H_2_O_2_ and O_2_^−^. Other probes for more specific ROS detection can also be incorporated into hydrogels. In that case, one should ensure that the probe is not reacting with the gel before plasma exposure or that the probe is not degraded during the hydrogel preparation process, for instance, during heating in the case of agarose or gelatin. The detection of hydrogen peroxide (H_2_O_2_) has been studied with Amplex UltraRed (AUR),^[^ ^141^, ^145^, ^181^, ^193^, ^194^ ^]^ titanium oxysulfate,^[^ ^195^ ^]^ OPD/HRP (o‐phenylenediamine dihydrochloride/horseradish peroxidase) or DCFH.^[^ ^196^ ^]^ However, this last probe was not specific to H_2_O_2_ compared to the previously mentioned probes.^[^ ^197^ ^]^ Production of nitrite (NO_2_^−^) is assessable by incorporating Griess reagent^[^ ^130^, ^141^, ^143^, ^181^, ^198^ ^]^ or sulfanilamide and N‐(1‐Naphthyl) ethylenediamine dihydrochloride.^[^ ^195^ ^]^ Peroxynitrite (ONOO^−^) reacts with folic acid^[^ ^195^ ^]^ or Dichlorodihydrofluorescein diacetate (H_2_DCF‐DA).^[^ ^145^ ^]^ Hydroxide (OH) presence is revealed by terephthalic acid^[^ ^195^, ^199^ ^]^ or 7‐hydroxycoumarin (7‐OH‐coumarin).^[^ ^141^ ^]^ The presence of aqueous O_3_ can be detected using indigo trisulfate.^[^ ^195^ ^]^ The very reactive nitric oxide (NO) is measured using the dye diaminofluorescein‐2 (DAF‐2).^[^ ^121^, ^145^ ^]^ Further probes such as Aminophenyl fluorescein (APF) for OH, HOCl ONOO^−^ and hydroxyphenyl fluorescein (HPF) for OH, ONOO^−^ encapsulated in collagen have been reported by.^[^ ^121^ ^]^ The main drawbacks of using such probes are probably the high reagent cost, suggesting small samples for sparing, combining the excellent sensitivity of the probes, and keeping the integrity of the gel. For instance, the gelation of some hydrogels can be problematic in the presence of acidic environments during cross‐linking.^[^ ^200^ ^]^ As a complement to ROS evaluation, pH reagents also give essential information on the plasma effect on tissues since plasma also has an acidic impact on surfaces.^[^ ^201^ ^]^ For this matter, investigations employing fluorescein,^[^ ^36^, ^202^ ^]^ or phenol red^[^ ^121^ ^]^ showed significant acidification of the gel after plasma treatment. They thus pointed out the need to anticipate such effects when targeting pH‐sensitive tissues.

Considering the question of penetration depth, some experiment designs use hydrogels as tissue or, more specifically, skin surrogates to investigate the role of an upper layer (i.e., skin) in ROS diffusion. Previously, it was shown that short‐lived species such as OH could not pass through the 4 mm thick hydrogel layer while a significant concentration of H_2_O_2_ was still present in the liquid below.^[^ ^203^ ^]^ By scavenging plasma‐generated OH radicals with the gel layer, this study also proved the impact of OH on DNA damage, envisaging hydrogel to protect open wounds from undesired species during plasma treatment. In previous work, a tissue model gel was also disposed of upon a ROS reactive hydrogel layer to quantify penetration depth and ROS diffusion pattern.^[^ ^189^, ^204^ ^]^ The molding of hydrogel offers matrices with creative forms that can mimic internal parts of the body, such as veins or intestines. In another study, agarose with ROS‐fluorescent reagent was molded in either an L or T shape matrix to observe the diffusion in cavities.^[^ ^194^ ^]^ The arrival of 3D‐printed scaffolds may enhance this method and offer even more complex networks. Plasma devices can be investigated and tested through hydrogel models, where parameters such as treatment mode, gas flow, and electric field can then be studied.^[^ ^182^ ^]^ In previous work, the hydrogel is directly used as a surrogate for nail simulation to evaluate the efficiency of a single‐use DBD patch electrode.^[^ ^134^ ^]^ In plasma medicine, it has been shown that biological effects are significantly driven by interactions with plasma‐derived ROS.^[^ ^18^, ^126^, ^205^, ^206^ ^]^ In this regard, comparable to ROS effect assessment, hydrogels are suitable for translating plasma‐physical impacts on bio‐materials and targeting retroaction onto plasma.^[^ ^194^ ^]^ However, more studies need to be done on this matter, as discussed in the prospects and developments section. It is also worth noticing that the hydrogel approach contributes to avoiding the extensive use of animals for experiments, which is not negligible for ethical research. Overall, hydrogels appeared to effectively serve as a bridge between in vivo and 2D models in plasma research applied to biology, offering a multitude of creative setups.

## 5 Prospects and Developments of Hydrogel in Plasma Research Applied to Medicine

Translational research aims to develop new technologies that directly impact and promote human welfare. Derived from this, several types of studies can be conducted, such as proof of concepts, animal studies, or clinical trials, to determine the potential of use and assess safety, efficacy, and suitability. In preclinical plasma medicine, hydrogels can be helpful in tissue engineering. Still, challenges remain in the translational relevance of the different hydrogels used as tissue surrogates and the influence of their composition. In addition, suitable methods to address ROS distribution and the contribution of other plasma effectors on biological tissues have to be further established. Hydrogels and cold physical plasma are already established or at least closely investigated for therapeutic purposes regarding wound healing or, more recently, cancer therapeutics. At the same time, the symbiosis of both seems to be on its way. However, future applications in clinics of plasma‐treated hydrogel, e.g., regenerative medicine, require their approval through clinical studies, which is costly and time‐consuming. Preclinical proof‐of‐concepts have successfully demonstrated the feasibility of this approach. However, approaches and aims for implementation in clinical settings are still under discussion. Recently, studies have explored injectable plasma‐treated hydrogels, e.g., for osteosarcoma treatment.^[^ ^142^, ^207^ ^]^ There are numerous strategies, and collaborations with clinicians are highly valuable.

**Figure 11:**
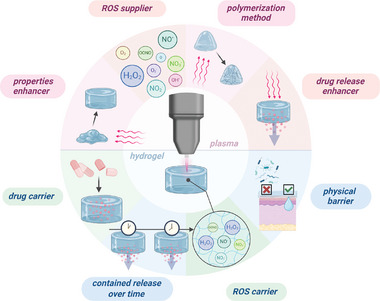
Potential symbiosis between cold physical plasma and hydrogels. Benefits offered by plasma technologies for enhanced hydrogel uses (pink top) along with hydrogel intrinsic properties for synergetic outcomes. Copyright: the authors.

In this combination, both hydrogel and plasma play a multipurpose role (**Table** **4**). Through heat, UV, and ROS production, plasma can polymerize the hydrogel, bringing various advantages. Plasma avoids using toxic chemicals for polymerization while supplying the hydrogel with ROS like H_2_O_2_. Plasma‐induced cross‐linking is efficient in contacting gels' main weakness, i.e., mechanical properties. Swelling properties were also improved by plasma treatment for a tyramine hyaluronic acid combined gel^[^ ^208^ ^]^ or gelatin‐hyaluronic acid gels.^[^ ^209^ ^]^ This translates to the ability of hydrogels to store the plasma‐derived ROS while it is being polymerized. When hydrogel is loaded with either drugs or cells, plasma operates as a switch on the gel by inducing stimuli. This is made possible mainly by developing “smart” hydrogels.^[^ ^97^ ^]^ For drug release, local plasma treatment could allow molecules in the gel to be delivered to wounds or tumors.^[^ ^100^, ^209^, ^210^ ^]^ In vitro, the plasma effect is dose‐dependent. In one case, plasma can enhance cell proliferation and contribute to the repopulation of the matrix. However, the opposite effect was observed at a distinct dose, inhibiting cell growth.^[^ ^145^ ^]^ The biological effects of plasma induced by ROS chemistry have been widely investigated; however, effects caused by plasma physical properties such as UV or electric field on biological matrices remain timidly examined and could deserve more attention. Previously, the generation of UV‐produced ROS at the surface of the target was shown in addition to the usual plasma‐delivered ROS.^[^ ^211^ ^]^ All tissues contain a minimum water content of ≈40% and can reach up to ≈90%.^[^ ^172^ ^]^ Therefore, they are intrinsically conductive. Furthermore, the state of plasma, however electrically neutral, can interact with surrounding targets if electrically non‐inert, as proven in,^[^ ^212^ ^]^ where a conductive treatment configuration was studied. The impact of plasma‐induced electric field and frequency on hydrogel matrices was shown in,^[^ ^145^, ^213^, ^214^ ^]^ highlighting primarily a target thickness dependency. Additional E‐field applied during plasma treatment showed a significantly deeper ROS penetration, especially for higher gelatin with higher water content.^[^ ^215^ ^]^ In those domains, further research would be beneficial in the future to better understand plasma‐tissue interactions (**Figure** **11**).

**Table 4:** Symbiosis of cold physical plasma and hydrogels. Copyright: the authors.

Therapeutic purpose	Hydrogel type	Hydrogel adjuvant	Plasma source	Main findings	Refs.
Cancer	Hyaluronic acid	Tyramine,ß‐blocking nano adjuvant TLN	FLA Medic+	Cold physical plasma used for cross‐linking ((Tyramine‐HRP)+HA combined with TLN) once injected into the tumor	[208]
Chronic wounds	Pyrrole (Ppy), hyaluronic acid (HA), gelatin (GEL)	Therapeutic platelet proteins	S‐DBD (argon)	Plasma for cross‐linking, enhanced mechanical features, slow therapeutic release, retention effect, NIR‐driven hyperthermic effect on lesion (heat shock proteins)	[209]
Wound (burn), antibacterial	Hyaluronic acid	Cur‐ZIF8@HA	Plasma jet (helium)	Burn treatment, combined treatment with loaded drug hydrogel and direct plasma treatment, gel pH sensitive to cold physical plasma	[262]
Cancer	Collagen	–	Plasma jet (helium)	Investigation of electric fields and plasma frequency in collagen hydrogel during plasma treatment	[214]

## 6 Conclusion

This work summarized the potential of hydrogels for biomedical purposes like wound healing, regenerative medicine, drug delivery, 3D cellular biology, or even for developing biosensors, all of which can be combined for specific applications. The design of hydrogels is headed to improve their mechanical properties and general biocompatibility. In this regard, well‐known gels like collagen have been closely investigated, but further investigation into new hybrid hydrogels would be helpful. On the other hand, plasma technologies have been given more consideration over the past decades in the medical field, notably wound healing. The combination of plasma and hydrogels comes in several forms. The enhancement of hydrogel properties could be enabled by multiple plasma parameters. Using hydrogel as a tissue surrogate helps to unravel the fundamental mechanisms of plasma‐tissue interactions. Several methods have been established, admitting there is still room for improvement. In the meantime, recent outcomes are encouraging for basic or translational medical research.

## Conflict of Interest

The authors declare no conflict of interest.
